# P-230. Frequency and Outcomes of Coagulase-Negative Staphylococcus Isolation from Blood Cultures of Patients Receiving Extracorporeal Membrane Oxygenation

**DOI:** 10.1093/ofid/ofae631.434

**Published:** 2025-01-29

**Authors:** Christian Wells, Erika O’Neil, Michal Sobieszczyk, Joseph Marcus

**Affiliations:** Brooke Army Medical Center, San Antonio, Texas; Brooke Army Medical Center, San Antonio, Texas; Brooke Army Medical Center, San Antonio, Texas; Brooke Army Medical Center, San Antonio, Texas

## Abstract

**Background:**

Coagulase-negative Staphylococcus (CoNS) are frequently cited as the most common cause of bacteremia in patients receiving extracorporeal membrane oxygenation (ECMO). CoNS bacteremia is concerning in ECMO as the biofilm it forms can adhere to the circuit. Alternatively, CoNS can be colonizing or contaminants, and there are no standard definitions of CoNS bacteremia in ECMO. Additionally, appropriate workup and follow-up for CoNS bacteremia in ECMO patients is unknown. This study evaluates the etiology and workup of CoNS in a cohort of patients receiving ECMO at a military medical center.

**Figure:**
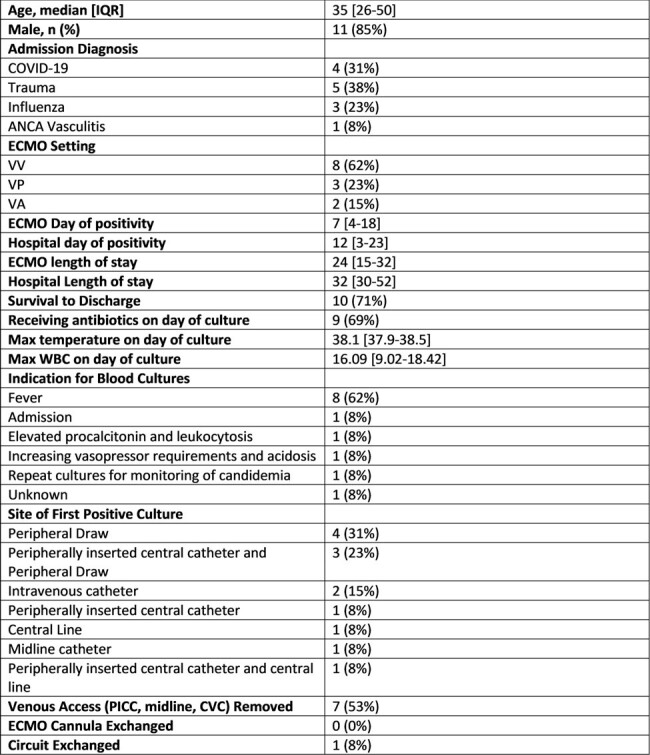

Characteristics of the 13 patients receiving ECMO with Coagulase-negative Staphylococcus isolated from blood cultures

**Methods:**

All patients who received ECMO between January 2022 and March 2024 were included in this retrospective study. All blood cultures were reviewed and patients with a positive blood culture for CoNS were included for further analysis including patient demographics, indication for culture, follow-up of culture results, treatment, and patient outcomes.

**Figure:**
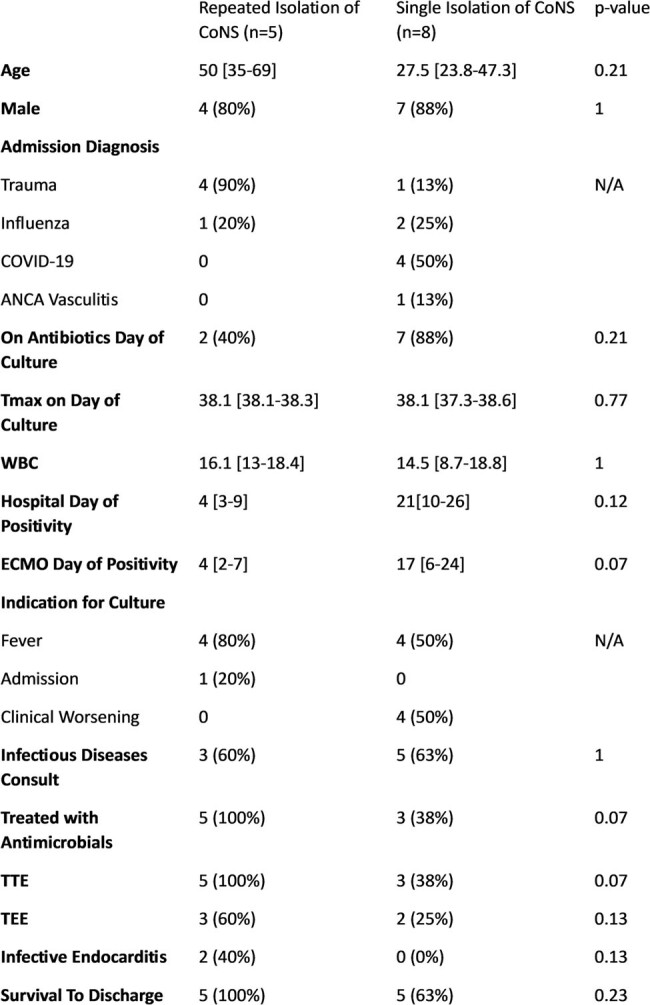

Characteristics of CoNS with Repeated Blood Culture Positivity as Compared to Single Blood Culture Positivity in Patients Receiving ECMO.

**Results:**

Of the 68 patients who received ECMO during the study period, there were 424 blood culture sets obtained, of which 20 (4%) yielded CoNS in 13 (19%) patients. Patients were predominantly male (85%) with a median age of 35 [IQR: 26-50] (**Table 1**). The most common indication for culture in this cohort was fever (62%) and most cultures were drawn from peripheral draws. All CoNS were monomicrobial and *S. epidermidis* (85%) accounted for the majority of cases. Only 5 (38%) patients had repeat growth of CoNS on repeat cultures, yet 8 (62%) were treated with antimicrobials (**Table 2)**. Two (15%) patients, both with persistent culture positivity, were found to have infective endocarditis. Of patients with multiple isolations of CoNS, all survived to hospital discharge. No patient had reisolation of CoNS after treatment.

**Conclusion:**

CoNS was frequently isolated in blood cultures from patients receiving ECMO. Despite the concern for biofilm formation, most CoNS was only isolated in one culture, and bacteremia was not associated with increased mortality, even when isolated repeatedly. Based on this data, it is reasonable to recommend treatment of CoNS bacteremia, only when isolated on repeat cultures. Standardization of CoNS bacteremia reporting, in addition to treatment in ECMO is needed.

**Disclosures:**

**All Authors**: No reported disclosures

